# Attempting to explore chiropractors and their clinical choices: an examination of a failed study

**DOI:** 10.1186/s12998-019-0236-0

**Published:** 2019-04-03

**Authors:** Stanley I. Innes, Charlotte Leboeuf-Yde, Bruce F. Walker

**Affiliations:** 1 College of Science, Health, Engineering and Education, Murdoch University, Murdoch, Australia; 2 Institute for Regional Health Research, University of Southern Denmark, DK-5000 Odens, Odense, Denmark

**Keywords:** Chiropractic, Clinical decisions, Big 5 personality, Intolerance of uncertainty, Subluxation, Philosophy

## Abstract

**Background:**

Recent studies have shown that psychological factors, attitudes and beliefs impact on the quality of chiropractic student clinical decisions. This association has not been studied among qualified chiropractors. Our objective was to investigate if personality, psychological factors and/or unorthodox beliefs among chiropractors are related to choices of management in specific clinical scenarios.

**Method:**

In February 2018, a subsample of chiropractors (*N* = 700) from a practitioner-based research network in Australia known as ACORN (*N* = 1680), were invited to respond to an on-line anonymous questionnaire. Questions included items relating to management of specific clinical scenarios, intolerance of uncertainty (IU) and the ‘Big-5’ personality score, adoption of a prescriptive technique system, self-rating of chiropractic abilities, and the level of importance of subluxation and chiropractic philosophy in the delivery of care. Descriptive analysis was to be reported and associations examined between i) personality and psychology factors, unorthodox beliefs and ii) scores obtained for management of specific clinical scenarios, numbers of interdisciplinary referrals, and guideline-based X-ray use.

**Results:**

The number of respondents was 141 (20%) and 33 of their responses were largely incomplete resulting in a final response rate of 108 (15.4%). In addition, some questions were left unanswered. These related mainly to IU and Big-5 personality measurements. Some sample characteristics (age, number of patients per week, hours worked per week) were similar to the larger ACORN project sample. However, the low response rate indicated that the final study sample was unlikely to be truly representative of the study population and the low number of participants made association testing unsuitable.

**Conclusion and recommendations:**

The low response rate and small study sample in this study made any substantive analysis inappropriate. For these reasons, the study was not concluded. However, the potential reasons for the low response from this large database of volunteer research participants are of interest and need to be investigated. Clearly, it is necessary to engage this population better to explore sensitive issues such as personality inventories and different practice profiles in the interest of effective health care delivery and patient safety.

**Electronic supplementary material:**

The online version of this article (10.1186/s12998-019-0236-0) contains supplementary material, which is available to authorized users.

## Background

The recognition of the adverse impact that persistent lower back pain (LBP) places on society has been evoked so often that it would appear to be verging on a cliché. The metrics on the extent of this large problem have been regularly and recently detailed [1]. Thus it is generally accepted that LBP is the leading cause of disability worldwide, and it is estimated to affect 540 million people at any one time [1]. This has culminated in a call for action to try to halt the increasing burden it imposes on the healthcare system [2]. As part of this call, a review of the current state of the prevention and treatment of LBP called for [3], among other strategies, the cessation of low value care and clear pathways for referral for the delivery of the correct treatment at the right time and the integration of traditional healers into the health-care system. Some chiropractors have sought to position themselves as spinal care specialists [4] and the foundational basis of this is that chiropractors should be making clinical decisions that result in the delivery of efficient high value care.

### Factors that impact on chiropractic decision making

Clinician decisions among first contact health practitioners have been described as being sometimes difficult to comprehend, because they do not always appear to follow logical pathways of reasoning [5–7]. Poor chiropractic clinical decisions have been identified as consequences of belief systems [8]. Some chiropractors align themselves with a vitalist philosophy, described by some as ‘unorthodox’ [9], and perceive that the ‘subluxation’ is a cause of disease that can be remedied with a spinal manipulation or ‘adjustment’ [9]. This group has demonstrated a predilection for non-guideline use of X-rays, non-evidence based treatment choices, and a lower likelihood of engaging in inter-professional collaboration [9].

Clinical decision-making has recently been studied in chiropractic students [10, 11]. These students appear to struggle recognising non-indicated care [11] (Goncalves et al., in press) and this difficulty was also extremely strongly associated with unorthodox beliefs (Goncalves et al., in press).

One of these studies of chiropractic students has shown a relationship between decision making and psychological factors such as intolerance of uncertainty (IU) [10]. IU refers to a dispositional characteristic that reflects a set of negative beliefs about uncertainty and represents an underlying fear of the unknown [12, 13]. These negative beliefs result in a desire for predictability [14, 15] and are associated with lower levels of confidence with decision-making [13, 16]. For medical practitioners, IU has been shown to be associated with a range of less than desirable outcomes such as lower compliance with evidence-based guidelines, [17] and generally increased resource use in the health care system [18]. The role of IU has not been explored in practising chiropractors’ clinical decisions.

This recent study exploring IU in chiropractic students found that the vast majority of students had the desire to learn a prescriptive system that would indicate ‘where the problem is’ and ‘how to treat it’, rather than accepting the grey shades of clinical reality [10]. However, unexpectedly, IU was not found to be a significant predictor of this desire and this was thought to be due to a lack of clinical experience or that the IU failed to capture a relevant psychological profile. The authors of that study suggested that further research with practicing chiropractors adding broader psychological measures and questions with respect to technique adoption might bring more information. The “Big 5” psychological traits / dimensions, also known as the Five Factor Model, is a widely accepted and commonly used model of personality [19, 20] and, as such, may be an appropriate broader measure of personality to further study this unexpected result.

While practitioner uncertainty and anxiety may impact negatively on clinical decisions [21, 22] over self-confidence has been shown to be equally detrimental in medical students and physicians [23]. This also has not been studied in chiropractors.

Thus, we wanted to understand better how the psychological profiles of chiropractors might impact on their health care decisions. To this end, and based on previous research findings, we proposed four different frameworks of conceptualizing how a chiropractor may be influenced, when making clinical decisions by examining:

- Intolerance of Uncertainty (IU)
- Unorthodox / Vitalist belief systems
- Self-rating / Overconfidence
- The “Big 5” psychological profiles of agreeableness, conscientiousness, extroversion, negative emotions, and openness to experience.

Our objectives were to determine if categorising chiropractors according to these frameworks would reveal differing responses on the following aspects of health care decisions:

1. Selecting appropriate care (Indicated / Non-indicated / Contra-indicated care case scenarios)
2. X-ray usage (number of new patients X-rayed)
3. Inter-disciplinary collaboration (percentages of Formal / Informal Medical Practitioner referrals made in the past week)
4. Demographic variables (patient numbers, number of hours worked, years in practice)
5. Adoption of a prescriptive ‘technique system’ for determining how to ‘locate’ and treat back pain
6. X-ray utilisation for determining how to locate and treat back pain i.e. non-guideline-based usage.

## Methods

### Procedure

In March 2018 our questionnaire was distributed via an e-mail invitation to 700 chiropractors who were members of the Australian Chiropractic Research Network (ACORN) practitioner cohort [24]. This was a quantitative cross-sectional descriptive study using an anonymous on-line survey platform (Survey Monkey).

Before distribution, the survey was first pilot tested on a small number of practitioners and academic staff for errors and to measure the time taken for completion. This resulted in some minor sentence structure changes. The initial invitation e-mail contained details of the study, the involved researchers’ names as well as information that the likely completion time was 25 to 30 min. A follow-up reminder e-mail was sent 4 weeks after the initial distribution. This was the maximum number of reminders allowed by ACORN.

The ACORN consists of a practitioner cohort of 1680 chiropractors, which represented approximately 36% of the licensed chiropractors in Australia, who were recruited from a national survey of all chiropractors in 2015. These chiropractors have since been approached in several surveys (but not all yet published) and to not fatigue the ACORN participants, questionnaires are sent out only to a randomly selected proportion of the cohort for each study.

Human Research Ethics approval was granted by Murdoch University (Project No 2017/157).

### The questionnaire (see Additional file 1)

Information on the demographic details of the participants and their clinics was sought (sex, age, years of practice, number of patients, and X-ray usage).

Two basic cases with several potential scenarios were presented (Additional file 1) seeking chiropractors’ clinical decisions in relation to a patient with neck pain and another with low back pain. The neck and low back pain (LBP) questionnaires have been previously used to assess chiropractors’ clinical decision-making profiles [25–28].

#### Neck pain

A neck pain case study with five scenarios, beginning with a simple uncomplicated case of neck pain and progressing through to a scenario requiring immediate medical referral was presented [26]. The case study was designed so that it could be used to differentiate between chiropractors who select appropriate / inappropriate and indicated / non-indicated or contra-indicated intervention strategies and has been described elsewhere [11, 26].

In brief, we replicated a previous study where scenarios 1 and 2 of the case study were designated as the ‘indicated’ or ‘correct’ choice [11]. These patient scenarios described simple uncomplicated neck pain and the ‘appropriate’ or ‘indicated’ choice was to treat, for both.

In addition, we selected scenario 5 as an example of a ‘contraindicated’ case. In this scenario the patient with neck pain had been resistant to treatment and there was clear evidence of progressive neurological deterioration. Selection of any option other than the referral choice was deemed to be ‘inappropriate’ because ‘contraindicated’ (the explanation and validation is seen in Additional file 1).

#### Low back

The second case described a range of clinical scenarios for a patient with low back pain designed to find out which management strategies chiropractors would prefer to use [25] (Additional file 1). This questionnaire had nine possible short term outcomes that were briefly described. Six clinical management alternatives were offered for each outcome scenario, going from treatment, external opinion and/or assistance, to referral out. The details for the design of this case study, validation and subsequent research are provided elsewhere [11, 25, 27, 28].

We selected three scenarios (1, 4, 8 and 9) for this study (Additional file 1).

Scenario 1 describes a first episode of uncomplicated LBP with rapid and complete resolution and, therefore, on-going care was regarded as ‘non-indicated’, with the correct decision being patient discharge. In scenario 9, the patient is non-responsive to treatment and is displaying signs of depression. Any continued treatment was regarded as ‘non-indicated’, i.e. of no avail but not potentially damaging.

Scenario 4 describes a patient with LBP who improves with treatment with a history of some previous uncomplicated episodes of acute LBP where each prior bout had completely resolved. The ‘indicated’ correct choice is some form of ‘maintenance care’.

The patient in Scenario 8 is resistant to treatment and is getting worse. A second opinion is required and continued care would be ‘contra-indicated’.

#### Psychological measurements

##### Intolerance of uncertainty scale (IUS-12)

To study the intolerance of uncertainty, we used the validated 12-question version (IUS-12) that utilises a 5-point Likert scale with responses ranging from ‘not at all characteristic of me’ to ‘entirely characteristic of me’ [12, 29–32]. Examples of questions are ‘unforeseen events upset me greatly’ and ‘the smallest doubt can stop me from acting’. The maximum possible score is 60, reflecting high levels of intolerance of uncertainty.

##### Big five Inventory-2 (BFI-2)

The Big Five Inventory-2 is a 60 item inventory designed and validated to measure the five personality dimensions of agreeableness, conscientiousness, extroversion, negative emotionality and open-mindedness [33]. Responses utilise a 5-point Likert scale and range from ‘disagree strongly’ to ‘agree strongly’.

#### Other measurements

##### Adoption of chiropractic technique system of analysis

Nine chiropractic technique systems were listed (See Additional file 1). These were selected because thought by the authors to be the most commonly used technique systems, which offer a method of identifying and correcting spinal dysfunctions. Participants were asked to select the degree to which they would use each techniques system’s method for analysing and / or guiding patient care. Responses ranged from ‘yes, as best I can’, ‘parts of it’ to ‘not at all’.

##### Unorthodox beliefs

Practitioners were asked to rate the importance of chiropractic philosophy and subluxation theory by asking the question: “How important is subluxation / chiropractic philosophy in what you do in practice?” We used a 5 point scale where possible responses ranged between ‘not at all important’ to ‘very important’.

##### Purpose of X-ray

Participants were also asked to indicate “yes” or “no” to a list of eight possible reasons why they might order X-rays. The options were: assessing for trauma, red flags, osteoporosis, osteoarthritis, spinal curves, contra-indications to chiropractic care, identifying subluxations, and assessing for patient progress.

##### Number of formal and informal referrals to a medical physician or specialist

Practitioners were asked to state what percentage of patients over the past week they would have formally (written letter or phone call) and informally (instructed the patient verbally) referred to a medical physician or medical specialist.

##### Self-rating

Finally, participants were asked to compare themselves as a chiropractor to other chiropractors in Australia.

### Variables of interest

#### Potential predictor variables

##### IU12

Receiver operating characteristic curves (ROC) were to be determined to find cut-off points in order to create high and low IU groups from the raw IU scores [34, 35]. This was to be calculated in order to evaluate potential high IUS-12 from the total IUS-12 score. Sensitivity and specificity of the coordinate points of the resulting ROC curve were then to be used to identify the potential cut-off score for IUS-12. This decision was also to be supported by previous published research with non-clinical samples that used similar values [34, 35].

##### Subluxation belief

The questions on subluxation belief was to be dichotomised with one group consisting of those who responded that subluxation theory was “important” or “very important” in guiding what they do in practice into one group vs. a group consisting of all the remaining responses (I have no opinion, neutral, only somewhat important, not at all important).

##### Chiropractic philosophy belief

In the same manner, chiropractors who responded that chiropractic philosophy was “important” or “very important” in what they do in practice would be compared with those who selected any other option.

##### Self-rating as a chiropractor compared to other chiropractors

Participants were asked to rate their professional level as a chiropractor in comparison to other chiropractors in Australia. Six options were possible ranging from “below average” to “above average”. This group would be dichotomised into those who selected “A bit above average” / “above average” and those who selected “average”, “I don’t know”, “a bit below average” or “below average”.

##### Big-five factors (BFI-2)

The “Big Five” raw scores were to be recoded into the 5 domains of agreeableness, conscientiousness, extroversion, negative emotions and openness to experience using the SPSS syntax provided by the authors of the BFI-2 in order to create 5 continuous measures [33].

### Outcome variables and their rationale

From this survey, variables shown to be associated with undesirable chiropractic practice characteristics were selected from previous research (as described above), in addition to the demographic and practice data:

- Indicated / non-indicated / contra-indicated care: Seven dependent variables were selected, three from the neck pain and four from the low back pain questionnaires. Of these two were *contra-indicated* cases, two *non-indicated* cases and three *indicated* cases. The ‘appropriate’ answers proposed in the previous study on this topic for the low back and neck questions were used [36]. The rationale is presented in Additional file 1.
- X-ray usage: The number of X-rays requested for the last 10 new patients would be split into 2 groups using the median score. This would potentially result in a ‘low’ and ‘high’ group for ordering X-rays.
- The number of informal and formal referrals made in the last full working week: Again we intended to create 2 groups (High, Low) on the basis of dichotomising the group on a 50% split, using the median score.
- Identification with need for technique system: Participants would be dichotomised into those who used a chiropractic technique system to guide clinical care (“Yes, as best I can” and “Parts of it”) and those who did not (“Not at all” and “the technique but not the system”).
- Reasons for taking X-rays: Respondents were to be dichotomised into those who ordered X-rays for a specified clinical reason and those who did not.

### Analysis

Data were to be summarised using descriptive statistics and reported as means, standards deviations or medians and IQRs, depending on normality for continuous data and frequency distributions for categorical data. Data would thereafter be transformed according to the previous descriptions.

Univariate group comparisons were to be made using chi-squared or Fisher Exact tests, as appropriate, for categorical data and t-test/ANOVA/Mann-Whitney U tests for continuous data. Factors associated with *IU-12, Subluxation and Philosophy beliefs, Self-rating and Big 5* binary outcomes would be explored using bivariate and multivariate logistic regression, with results expressed as odds ratios and their 95% confidence intervals.

Data were to be analysed using SPSS v.24 (IBM Corp, Armonk NY, USA).

## Results

### Descriptive information

Of the 700 contacted chiropractors, 141 responded to the on-line survey (20% response). The average time taken to complete the survey was 23 min.

However, there were 33 incomplete sets of data that resulted in a final 108 complete responses (15.4% response rate) of which 66% were males with a mean age of 44.3 (SD 11) years (Table 1). The 33 incomplete questionnaires all lacked the responses for practice characteristics, and the measures of IU and Big-5. Of these 33, only 4 responded to the neck and LBP scenarios although they all responded to questions on chiropractic philosophy, subluxation, and technique use.

**Table 1:** Demographic and practice variables of chiropractors from this study compared to the total ACORN chiropractor population [24]

Demographic Variable	Current Study	ACORN Cohort
Age: Mean (SD)	44.3 (11) Range 25–70	41.9 (12)
Male / Female:	66.1%	62.9%
Years in practice: Mean (SD)	17.9 (10.4) Range 2–44	15.6 (11.2)
Hours / Week Work: Mean (SD)	27.1 (10.3) Range 5–60	27.3 (12.8)
Patients / Week: Mean (SD)	91 (58.7) Range 8–310	87.5 (56.3)

This low response rate indicates that the study sample is unlikely to be representative of its study population. Nevertheless, the profile of the responders is presented in Tables 1 and 2 and summarized below.

**Table 2:** Construction of variable groups and their characteristics

Domains	Variable	Variable construction	Number (%)
Patients / week *Missing 39*	Mean 91.4 (58.7)	Low	27 (26)
	Median 82.5 / week	Average	49 (45)
	Range 7–310	High	26 (28)
*X-rays (High and Low)* *Missing 33*	New Patient X-rays,		
	Mean 3.6 (2.7)	Low	52 (48)
	Median 3,	high	41 (52)
	Range 0–10		
*Reason X-rays*	Contraindications	Yes	79 (73)
	*Missing 39*	No	29 (27)
	Osteoarthritis	Yes	70 (66)
	*Missing 35*	No	36 (43)
	Osteoporosis/paenia	Yes	63 (61)
	*Missing 38*	No	40 (39)
	Patient progress	Yes	5 (4)
	Missing 2	No	135 (96)
	Red Flags	Yes	98 (91)
	*Missing 39*	No	10 (9)
	Scoliosis	Yes	66 (61)
	*Missing 33*	No	42 (39)
	Subluxation	Yes	15 (11)
	*Missing 1*	No	125 (89)
	Trauma	Yes	99 (91)
	*Missing 32*	No	10 (9)
*Referral*			
Informal	Mean 4.9 (4.9)	No Referrals	50 (52)
(*N* = 108) *Missing 33*	Median 4,	At least one	45 (48)
	Range 0–40		
Formal	Mean 2.3 (2.7)	No Referrals	9 (8)
(*N* = 108) *Missing 33*	Median 2,	At least one	99 (91)
	Range 0–20		
*Technique*	Advanced Bio-structural	Yes & parts of it	7 (7)
	Correction	No to all else	89 (93)
	*Missing 45*		
	Activator	Yes & parts of it	32 (26)
	*Missing 17*	No to all else	92 (74)
	Applied Kinesiology	Yes & parts of it	42 (43)
	*Missing 19*	No to all else	80 (66)
	Chiropractic BioPhysics	Yes & parts of it	12 (11)
	*Missing 36*	No to all else	93 (89)
	Functional Neurology	Yes & parts of it	58 (51)
	*Missing 27*	No to all else	56 (40)
	Gonstead	Yes & parts of it	45 (40)
	*Missing 27*	No all else	69 (60)
	Neural Organisation Technique	Yes & parts of it	7(7)
	*Missing 38*	No to all else	96 (93)
	Sacro Occipital Technique	Yes & parts of it	58 (49)
	*Missing 23*	No to all else	60 (51)
	Thompson	Yes & parts of it	66 (54)
	*Missing 30*	No to all else	55 (46)
Chiropractic Philosophy	*Missing 8*	Very / Important	90 (68)
		All others	43 (32)
Subluxation	*Missing 1*	Very / Important	75 (53)
		All others	65 (47)
Self-rating	*Missing 37*	I don’t know / Av	29 (38)
		Above Av	75 (72)

The chiropractors in this study worked on average 27 h (SD 10.3) per week. They treated 91 (SD 58.7) patients per week and had been in practice for 17.9 (SD 10.4) years. These responses approximated those of the larger ACORN project participants whose characteristics are also shown in Table 1.

### Descriptive characteristics of clinical groups and subgroups

Almost 70% of the participants in this study regarded chiropractic philosophy as important or very important for what they do in practice (see Table 2). Half of the subjects rated subluxation as very important or important in guiding what they do in practice. Almost 75% of the chiropractors rated their professional level as a bit above average or above average.

### Associations between predictor and outcome variables

There were too few responders to make association testing appropriate.

## Discussion

### Summary of findings

This appears to be the first attempted study seeking to investigate chiropractors’ clinical reasoning. It sought to progress findings from previous studies in chiropractic student populations by proffering four different ‘psychological’ conceptual and belief frameworks that might influence clinical decisions that were, in turn, to be linked with a number of different aspects of chiropractic practice used as indicators of the quality of health care delivered.

However, only 141 chiropractors participated in this study and of these only 108 provided complete data; the final response rate therefore being only 15.4%. Although there were similarities with the larger ACORN population, this low response rate means it was not possible to generalize the findings of this study to the ACORN population and certainly not to the entire chiropractic population of Australia. The low number of responders made it unsuitable to perform tests of association.

### Explanation of findings

There is limited literature to guide researchers in the practical aspects of recruiting professionals for research studies. The magnitude of this problem has previously been demonstrated with a review of the National Institute for Health inventory of trials showing that only 34% of trials ever reached their projected sample size [37].

Nevertheless, previous studies of chiropractors in other countries have resulted in better response rates. For example; Denmark 72% [38], Finland 88% [39], Norway 61% [40], Sweden 60% [25] and 77% [41]. These studies were all conducted in Scandinavia. The low response rate in the present study could therefore be a cohort problem, particular to Australia, as only 43% of all registered chiropractors originally consented to become members of the ACORN ‘panel’. Of these 2005 practitioners, only 83% (1680) did thereafter agree to join. Despite this acceptance, the one other published study from this ‘panel’ reported a response rate of only 33% [42].

Even so, the response rate in our study is discordant with that previous ACORN study. There would therefore appear to be something that triggered a general non-response. The pilot testing did not identify any obstacles that can explain this low response rate.

A potential explanation for the difference in the willingness to participate in surveys could be that the Scandinavian studies might have been considered clinically ‘helpful’ as they related to clinical questions such as predictors of outcome and defining maintenance care. It is possible that the previous and present Australian studies were felt to be uninteresting and less meaningful. The additional level of disinterest in the present study could also have been in protest, if chiropractors felt the topic to be politically ‘dangerous’, dealing with practice behaviour, and perhaps too personal regarding personality traits.

Other explanations for potential participants choosing not to respond to such a degree that the study has ‘failed’ could include a lack of belief that a relationship existed between the variables being researched [43], an uncomfortableness with being seen as unable to manage the technical aspects of the study [44], the likely benefit of the study was not worth the time taken to respond [45], or that the research question was not ‘worthwhile’ [46]. It is not possible to definitively know which one, or which combination of these explanations – if any - impacted on this study.

Another significant impediment to response is likely to be the inability to remind chiropractors to respond. Best practice for enhancing response rates in surveys is to initially send a “herald” notice that the survey is coming and important, then a despatch of the survey and at least two follow-up reminders at spaced intervals [47]. Unfortunately, ACORN will not allow this type of reminder system and this could impair response rates in future surveys of ACORN participants.

Examination of the 33 incomplete response sets identified that absence of replies to the intolerance of uncertainty questionnaire (12 items) and the Big-5 inventory (60 items) was common to all of these partial responders. It is possible that non-responders failed to participate for the same or similar reason, namely, that this study gives the appearance of practice behaviour being associated with psychopathology or deviant behaviour patterns. As such, it could be negatively perceived as an ‘attack’ on the chiropractic profession. The offer of anonymity on the patient information recruitment email does not appear to have alleviated concerns.

Studies exploring psychological profiles and belief systems have been conducted in other health professions and have provided valuable insight into factors that impact on clinical decision making and have the potential to improve health economics and patient safety [17, 18, 23, 48]. These factors may also be important in chiropractic care. This study suggests that this is a ‘sensitive area’ and that future efforts to gather data to examine such issues may be inherently problematic. It is possible that repeated similar attempts will only result in accentuating this type of non-response behaviour and ‘drive it further underground’. One possibility to improve the situation may be to conduct a qualitative study by a known and trusted interviewer(s) to identify the reasons for this reluctance to participate. This method may also seek information from interviewees on ways to engage this population better. Once the drivers for this type of behaviour are identified it may be possible to better articulate the aims, wording and design of the study to potential participants and thus improve response rates. Past research has shown, with appropriate funding, the better known methods are monetary (preferably prepaid) and non-monetary incentives (lotteries, gifts) [49], SMS reminders [50], and personal contact to achieve this end [51]. Future studies may also benefit from adding locally known and trusted network leaders to the target population [52]. In addition, ACORN should review its restrictions on reminders to enhance response rates.

## Conclusions

This study was made untenable by a poor response making it impossible generalize any findings and to conduct any association testing. There are many possible explanations for this non-response. A way to engage this population to explore these poor practice profiles in the interest of effective health care delivery and patient safety is required.

## Additional file

Additional file 1: Survey Questionnaire and Rational for scoring of neck and LBP scenarios. The additional file contains the survey distributed to all of the ACORN practitioners. It also contains the rational for the scoring of the neck and LBP case scenarios as Contraindicated, Indicated and Non-indicated. (DOCX 366 kb)

## Abbreviations

ACORN: Australian Chiropractic Research Network
B.F.I-2: Big Five Inventory version 2
IU: Intolerance of uncertainty
S.D.: Standard Deviation
